# NHANES 2011–2014: Association Between Conicity Index and Cognitive Performance in Older Adults

**DOI:** 10.31083/AP46031

**Published:** 2025-08-25

**Authors:** Fei Chen, Ken Chen

**Affiliations:** ^1^Department of Geriatric, Chongqing Mental Health Center, 401147 Chongqing, China

**Keywords:** cognitive function, conicity index, cross-sectional survey, older adult, NHANES

## Abstract

**Background:**

The negative impact of obesity on cognitive function (CF) is well-established; nevertheless, no prior studies have explored the link between conicity index and cognitive performance. This research sought to investigate the link between conicity index and cognitive impairment.

**Methods:**

Data were obtained from a cross-sectional analysis of the National Health and Nutrition Examination Survey 2011–2014 (NHANES), with CF evaluated by the total scores of three cognitive tests (TCT), the delayed recall test (DRT), the immediate recall test (IRT), the animal fluency test (AFT), and the digit symbol substitution test (DSST). The conicity index was derived from waist circumference, height, and weight. Multiple linear regression, smooth curve fitting, and subgroup interaction analyses were utilized to explore the correlation between conicity index and cognitive performance.

**Results:**

The study included 2752 subjects and the results indicated that increasing conicity index was markedly associated with declining CF. In fully adjusted models, the conicity index was linked to reductions in total cognitive score (β = –16.35, 95% confidence interval (CI): –26.68 to –6.02, *p* = 0.0019) and DRT (β = –1.52, 95% CI: –2.74 to –0.30, *p* = 0.0151), IRT (β = –2.93, 95% CI: –5.37 to –0.48, *p* = 0.0190), AFT (β = –2.03, 95% CI: –4.88 to 0.82, *p* = 0.1636), and DSST (β = –9.88, 95% CI: –17.11 to –2.65, *p* = 0.0075) scores. However, the negative association between conicity index and AFT score was not statistically significant.

**Conclusions:**

Lower CF is associated with a higher conicity index. The conicity index is useful for the early detection of cognitive decline.

## Main Points

1. To investigate the link between the conicity index and cognitive function (CF) by cross-sectional study using NHANES data.

2. Lower CF is associated with a higher conicity index.

3. The conicity index proves beneficial for the early detection of cognitive decline.

## 1. Introduction

In recent decades, the prevalence and fatality rates linked to dementia have shown a consistent upward trend [1]. The World Health Organization estimates that approximately 50 million people worldwide are affected by dementia, with a new diagnosis occurring every three seconds. Projections indicate that by 2050, the number of individuals with dementia is projected to nearly double [2]. Dementia is primarily characterized by cognitive decline and reduced daily functioning, often accompanied by behavioral and psychological symptoms. These symptoms not only diminish the well-being of patients and their families but also impose significant psychological, financial, and caregiving burdens that contribute to an increasing strain on healthcare systems [3]. Cognitive impairment, particularly in the initial phases of dementia and Alzheimer’s disease, is a significant clinical manifestation. The progression from cognitive decline to Alzheimer’s disease is irreversible, and current pharmacological interventions remain ineffective in halting or improving its course. Identifying factors that influence cognitive function (CF) is therefore essential to developing preventive strategies.

Globally, obesity has emerged as a serious health concern and a recognized risk factor for various diseases, including metabolic disorders, cardiovascular diseases, osteoarthritis, dementia, depression, and cancer, all of which contribute to reduced life expectancy [4, 5, 6, 7]. While numerous investigations have explored the impact of obesity on cognitive impairment in older populations, no definitive conclusions have been reached. Inconsistent findings may be attributed, in part, to differences in the methods used to assess obesity. Among the various metrics, body mass index (BMI) is the most widely used, yet it fails to accurately reflect body composition, particularly in terms of fat and muscle distribution, and is unable to capture the distribution of adiposity [8, 9]. The conicity index, a novel anthropometric measure of obesity, takes into account an individual’s height, weight and waist circumference and calculates it through specific mathematical formulas, so as to obtain an index reflecting body shape and abdominal obesity. This index aids researchers to evaluate the degree of obesity from the perspective of weight distribution and body size, rather than relying on the traditional BMI. The conicity index is superior to general obesity indicators such as BMI in assessing the risk of diabetes and cardiovascular disease [10, 11]. At present, there is no study on the correlation between the conicity index and neurological diseases such as cognitive impairment. However, Diabetes mellitus and cardiovascular disease are also known to increase the risk of cognitive impairment and it is speculated here that the conicity index may have a correlation with CF. Hence, this investigation sought to examine the association between the conicity index and cognitive performance utilizing data from the National Health and Nutrition Examination Survey 2011–2014 (NHANES), and applying a multifactorial analysis of demographic characteristics and medical history.

## 2. Methods

### 2.1 Study Population

**Fig. 1.**
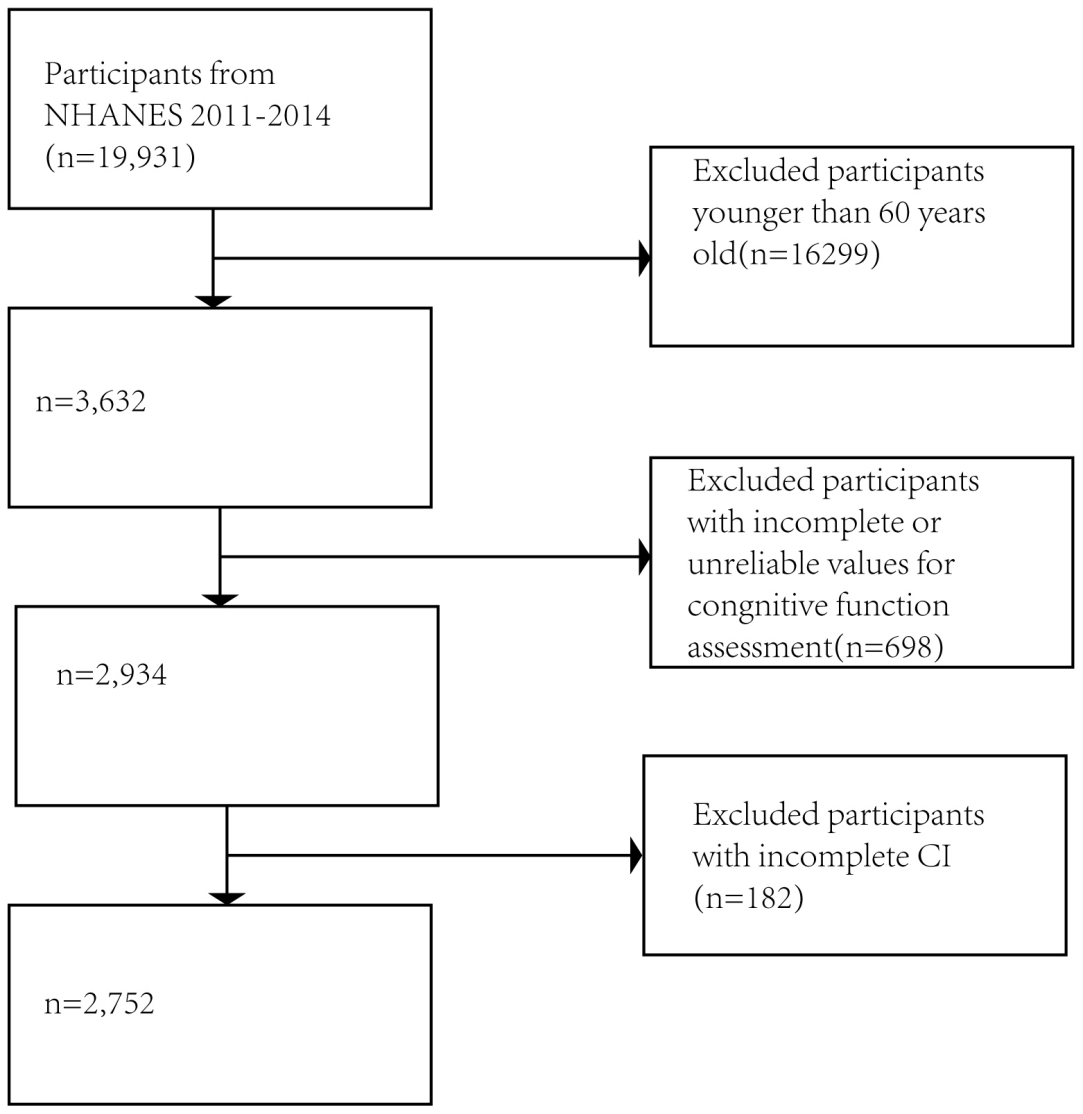
**Flow chart of the screening process for study population**.

The data utilized in this research were sourced from the NHANES database (http://www.cdc.gov/nchs/nhanes/), a cross-sectional survey aimed at evaluating the health status of the USA population. The survey was executed by the Centers for Disease Control and Prevention, and ethical approval was obtained for all NHANES protocols. Participants provided informed consent before their enrollment in the survey. NHANES is conducted biennially, in each cycle, through a complex multi-stage random sampling design, every year about 5000 people will be included in the survey research. The survey includes face-to-face interviews, physical examinations and laboratory examination. This study is a cross-sectional observational study that collected anonymous demographic and survey data from patients using the NHANES database. The years 2011–2012 and 2013–2014 were the two most recent survey periods with CF test scores, therefore data from these two survey periods was included in this study. A total of 19,931 individuals took part in NHANES from 2011 to 2014, with the current analysis restricted to 3632 participants aged 60 years or older. After excluding individuals with incomplete cognitive test data (n = 698) and missing conicity index values (n = 182), 2752 eligible subjects were incorporated in the final examination. Inclusion criteria: (1) Database survey data from 2011 to 2012 (*n* = 9756). (2) Database survey data from 2013 to 2014 (*n* = 10,175). Exclusion criteria: (1) Participants were younger than 60 years old (*n* = 16,299). (2) Participants did not have complete data for cognitive function measurements (*n* = 698). (3) Participants had no complete conicity index data (182) (Fig. 1).

### 2.2 CF Evaluation

CF in individuals aged 60 and above was assessed with three tests: (1) the Center for the Establishment of a Registry for Alzheimer’s Disease (CERAD) Word Learning Score Test (CERAD W-L); (2) the Animal Fluency Test (AFT); and (3) the Digit Symbol Substitution Test (DSST). The CERAD W-L test consists of three immediate recall tests (IRT) and one delayed recall test (DRT). In this test, subjects are presented three times with 10 unrelated words, and instructed to recall as many as possible after each presentation. Recall is delayed by approximately and follows the additional cognitive assessments. In the AFT, participants are allotted one minute to name as many animals as possible, receiving one point for each correct response. The DSST, adapted from the Wechsler Adult Intelligence Scale-III, requires individuals to pair corresponding symbols with numbers in 133 boxes within 120 seconds, with the final score reflecting the number of correct pairings.

In this study, the total scores from the three cognitive tests (TCT), as well as individual scores from the three IRTs, DRT, AFT, and DSST, were used as outcome variables. Since no definitive cut-off values exist for these cognitive measures, the 25th percentile of participants’ scores (the lowest 25th percentile) was adopted as the threshold for low CF, consistent with previous research practice [12]. The corresponding cut-off values for cognitive impairment in this cohort were 71, 16, 5, 13, and 34, respectively.

### 2.3 Conicity Index Assessment

The conicity index represents a novel indicator for assessing obesity, based on the concept that the human body undergoes a transformation from a cylindrical shape to a biconical form as abdominal fat accumulates. It is computed utilizing waist circumference, height, and body mass, with the formula:

$$\text{ Conicity index }=\text{ waist circumference }\,\left(m\right)/\left(0.109\times \sqrt{\text{ Weight }\,\left(\mathrm{kg}\right)/\text{ Height }\,\left(m\right)}\right)$$

[13]

In this study, waist circumference, height, and body mass measurements were acquired by qualified health specialists at the Mobile Examination Centers, and the conicity index was used as the primary exposure variable.

### 2.4 Covariates

To guarantee the precision of the findings, several covariates related to CF were included based on prior studies. These covariates encompassed age, sex (male or female), race/ethnicity (Mexican American, other Hispanic, non-Hispanic White, non-Hispanic Black, other races), educational attainment (below high school, high school, above high school), marital status (married, widowed, divorced, separated, unmarried, cohabiting), BMI, income-to-poverty ratio (IPR) the ratio of family income to poverty ≤1.3, >1.3, ≤3.0, >3.0), smoking habits (smoked at least 100 cigarettes in life or not), alcohol consumption (had at least 12 alcohol drinks per year or not), diabetes, and hypertension.

### 2.5 Statistical Analysis

Statistical analyses were performed using EmpowerStats (version 4.2) (http://www.empowerstats.net/analysis). In descriptive statistics, continuous variables are given as mean (± standard deviations), while categorical variables are given as frequencies and percentages. To assess differences between participants with low and normal CF, the Kruskal-Wallis rank-sum test was used for continuous variables, and Fisher’s exact test for categorical variables when the anticipated count was less than 10. Multiple logistic regression models were developed to investigate the link between the conicity index and impaired CF. Three models were tested: Model 1 included no covariate adjustments; Model 2 was adjusted for age, gender, education level, marital status, and race; Model 3 was further adjusted for IPR, BMI, smoking status, alcohol consumption, hypertension, and diabetes.

For missing data, continuous variables were imputed utilizing the mean, while categorical variables were grouped separately to account for missing values (the missing values of covariates education, marital status, IPR, alcohol consumption, hypertension, diabetes, smoking status were 2, 3, 231, 30, 4, 2 and 2, respectively and other variables had no missing values). And there are few missing values in this study, which is not the key indicator we focus on. The effect of missing values on study outcomes was not significant, so our results can be considered stable and trustworthy. The link between the conicity index and CF was visualized using smooth curve fitting, and interaction tests and subgroup analyses were conducted to verify the consistency of the observed relationship across different population subgroups. Statistical significance was established at *p* < 0.05.

## 3. Results

### 3.1 Baseline Characteristics

This investigation analyzed 1348 males and 1404 females from the 2011–2014 NHANES cohort, with an average age of 69.25 ± 6.73 years and a mean conicity index of 1.35 ± 0.08. The CF tests—TCT, DRT, IRT, DSST, and AFT—revealed that 26.09%, 39.64%, 27.07%, 25.98%, and 36.05% of participants, respectively, exhibited low cognitive performance. Significant differences in age, race, education level, IPR, and marital status were observed between individuals with low and normal CF across all five tests (*p* < 0.01). Participants with lower CF tended to be older, less educated, financially disadvantaged, widowed, and of non-Hispanic black ethnicity. In the low cognitive cohort, TCT, DRT, IRT, and DSST scores were markedly correlated with higher conicity index values (*p* < 0.01), while AFT scores were not correlated with the conicity index. Cognitive decline measured by TCT, DSST, and AFT was positively associated with the presence of diabetes and hypertension. According to DRT, individuals with poor cognitive performance were more likely to have diabetes compared to those with normal cognition. DRT revealed a higher prevalence of smokers in the low cognitive cohort (Table 1).

**Table 1.** **Characteristics of the study population, NHANES 2011–2014**.

		TCT			DRT			IRT			DSST			AFT		
Variables		Low	Normal	*p*-value	Low	Normal	*p*-value	Low	Normal	*p*-value	Low	Normal	*p*-value	Low	Normal	*p*-value
		cognitive Performance	cognitive performance		cognitive Performance	cognitive performance		cognitive Performance	cognitive performance		cognitive Performance	cognitive performance		cognitive Performance	cognitive performance	
N		718	2034		1091	1661		745	2007		715	2037		992	1760	
Conicity index		1.36 ± 0.08	1.35 ± 0.08	<0.001	1.36 ± 0.08	1.35 ± 0.08	<0.001	1.37 ± 0.08	1.35 ± 0.08	<0.001	1.36 ± 0.08	1.35 ± 0.08	<0.001	1.36 ± 0.08	1.35 ± 0.08	0.210
Age		71.54 ± 6.89	68.44 ± 6.49	<0.001	71.08 ± 6.89	68.06 ± 6.35	<0.001	71.47 ± 6.95	68.43 ± 6.46	<0.001	71.00 ± 6.85	68.64 ± 6.58	<0.001	70.39 ± 6.87	68.61 ± 6.56	<0.001
BMI		28.72 ± 6.06	29.07 ± 6.26	0.235	28.63 ± 5.96	29.21 ± 6.36	0.025	28.42 ± 5.90	29.19 ± 6.31	0.006	28.66 ± 5.96	29.09 ± 6.30	0.204	28.81 ± 6.27	29.08 ± 6.18	0.130
Sex, n (%)				<0.001			<0.001			<0.001			<0.001			0.639
	Male	402 (55.99%)	946 (46.51%)		639 (58.57%)	709 (42.69%)		456 (61.21%)	892 (44.44%)		404 (56.50%)	944 (46.34%)		480 (48.39%)	868 (49.32%)	
	Female	316 (44.01%)	1088 (53.49%)		452 (41.43%)	952 (57.31%)		289 (38.79%)	1115 (55.56%)		311 (43.50%)	1093 (53.66%)		512 (51.61%)	892 (50.68%)	
Race, n (%)				<0.001			<0.001			<0.001			<0.001			<0.001
	Mexican American	93 (12.95%)	156 (7.67%)		113 (10.36%)	136 (8.19%)		83 (11.14%)	166 (8.27%)		97 (13.57%)	152 (7.46%)		83 (8.37%)	166 (9.43%)	
	Other Hispanic	134 (18.66%)	153 (7.52%)		131 (12.01%)	156 (9.39%)		103 (13.83%)	184 (9.17%)		140 (19.58%)	147 (7.22%)		121 (12.20%)	166 (9.43%)	
	Non-Hispanic White	213 (29.67%)	1084 (53.29%)		497 (45.55%)	800 (48.16%)		320 (42.95%)	977 (48.68%)		191 (26.71%)	1106 (54.30%)		342 (34.48%)	955 (54.26%)	
	Non-Hispanic Black	232 (32.31%)	417 (20.50%)		275 (25.21%)	374 (22.52%)		172 (23.09%)	477 (23.77%)		240 (33.57%)	409 (20.08%)		316 (31.85%)	333 (18.92%)	
	Other race	46 (6.41%)	224 (11.01%)		75 (6.87%)	195 (11.74%)		67 (8.99%)	203 (10.11%)		47 (6.57%)	223 (10.95%)		130 (13.10%)	140 (7.95%)	
Education, n (%)				<0.001			<0.001			<0.001			<0.001			<0.001
	Below high school	394 (54.87%)	296 (14.55%)		383 (35.11%)	307 (18.48%)		310 (41.61%)	380 (18.93%)		409 (57.20%)	281 (13.79%)		370 (37.30%)	320 (18.18%)	
	High school	163 (22.70%)	478 (23.50%)		263 (24.11%)	378 (22.76%)		166 (22.28%)	475 (23.67%)		159 (22.24%)	482 (23.66%)		250 (25.20%)	391 (22.22%)	
	Above high school	159 (22.14%)	1260 (61.95%)		443 (40.60%)	976 (58.76%)		268 (35.97%)	1151 (57.35%)		145 (20.28%)	1274 (62.54%)		370 (37.30%)	1049 (59.60%)	
Marital Status, n (%)				<0.001			0.004			0.004			<0.001			<0.001
	Married	347 (48.33%)	1191 (58.55%)		584 (53.53%)	954 (57.44%)		397 (53.29%)	1141 (56.85%)		347 (48.53%)	1191 (58.47%)		540 (54.44%)	998 (56.70%)	
	Widowed	187 (26.04%)	320 (15.73%)		237 (21.72%)	270 (16.26%)		166 (22.28%)	341 (16.99%)		182 (25.45%)	325 (15.95%)		217 (21.88%)	290 (16.48%)	
	Divorced	86 (11.98%)	307 (15.09%)		140 (12.83%)	253 (15.23%)		88 (11.81%)	305 (15.20%)		84 (11.75%)	309 (15.17%)		122 (12.30%)	271 (15.40%)	
	Separated	39 (5.43%)	39 (1.92%)		34 (3.12%)	44 (2.65%)		27 (3.62%)	51 (2.54%)		42 (5.87%)	36 (1.77%)		37 (3.73%)	41 (2.33%)	
	Never married	42 (5.85%)	116 (5.70%)		61 (5.59%)	97 (5.84%)		42 (5.64%)	116 (5.78%)		41 (5.73%)	117 (5.74%)		56 (5.65%)	102 (5.80%)	
	Living with partner	17 (2.37%)	58 (2.85%)		35 (3.21%)	40 (2.41%)		25 (3.36%)	50 (2.49%)		19 (2.66%)	56 (2.75%)		19 (1.92%)	56 (3.18%)	
IPR, n (%)				<0.001			<0.001			<0.001			<0.001			<0.001
	≤1.3	322 (44.85%)	424 (20.84%)		354 (32.45%)	392 (23.60%)		273 (36.64%)	473 (23.57%)		320 (44.76%)	426 (20.91%)		341 (34.38%)	405 (23.01%)	
	>1.3, ≤3	208 (28.97%)	582 (28.61%)		334 (30.61%)	456 (27.45%)		214 (28.72%)	576 (28.70%)		219 (30.63%)	571 (28.03%)		284 (28.63%)	506 (28.75%)	
	>3	117 (16.30%)	868 (42.67%)		310 (28.41%)	675 (40.64%)		189 (25.37%)	796 (39.66%)		105 (14.69%)	880 (43.20%)		263 (26.51%)	722 (41.02%)	
Alcohol Consumption, n (%)				<0.001			0.859			0.440			<0.001			<0.001
	Yes	438 (61.00%)	1436 (70.60%)		738 (67.64%)	1136 (68.40%)		495 (66.44%)	1379 (68.71%)		439 (61.40%)	1435 (70.45%)		621 (62.60%)	1253 (71.20%)	
	No	264 (36.77%)	584 (28.71%)		337 (30.89%)	511 (30.76%)		236 (31.68%)	612 (30.50%)		260 (36.36%)	588 (28.87%)		355 (35.79%)	493 (28.01%)	
Hypertension, n (%)				<0.001			0.160			0.162			<0.001			<0.001
	Yes	483 (67.27%)	1213 (59.64%)		689 (63.15%)	1007 (60.63%)		475 (63.76%)	1221 (60.93%)		491 (68.67%)	1205 (59.27%)		660 (66.67%)	1036 (58.93%)	
	No	233 (32.45%)	819 (40.27%)		399 (36.57%)	653 (39.31%)		269 (36.16%)	783 (39.07%)		224 (31.33%)	828 (40.73%)		330 (33.33%)	722 (41.07%)	
Diabetes, n (%)				<0.001			0.012			0.055			<0.001			<0.001
	Yes	218 (30.36%)	415 (20.40%)		276 (25.30%)	357 (21.49%)		194 (26.04%)	439 (21.87%)		221 (30.91%)	412 (20.23%)		268 (27.02%)	365 (20.74%)	
	No	466 (64.90%)	1526 (75.02%)		757 (69.39%)	1235 (74.35%)		515 (69.13%)	1477 (72.10%)		460 (64.34%)	1532 (75.21%)		674 (67.941%)	1318 (74.87%)	
	Broadline	33 (4.60%)	92 (4.52%)		58 (5.32%)	67 (4.03%)		36 (4.83%)	89 (4.43%)		33 (4.62%)	92 (4.52%)		49 (4.94%)	76 (4.32%)	
Smoking status, n (%)				0.152			0.022			0.846			0.098			0.892
	Yes	381 (53.06%)	1017 (50.00%)		583 (53.44%)	815 (49.07%)		381 (51.14%)	1017 (50.67%)		382 (53.43%)	1016 (49.88%)		506 (51.01%)	892 (50.68%)	
	No	336 (46.80%)	1016 (49.95%)		506 (46.38%)	846 (50.93%)		364 (48.86%)	988 (49.23%)		332 (46.43%)	1020 (50.08%)		486 (48.99%)	866 (49.20%)	

Notes: The amount of missing values for the covariates were 2 (0.07%) for education, 3 (0.11%) for marital status, 231 (8.40%) for IPR, 30 (1.10%) for alcohol consumption, 4 (0.15%) for hypertension, 2 (0.07%) for diabetes, 2 (0.07%) for smoking status. Abbreviations: AFT, animal fluency test; BMI, body mass index; DRT, delayed recall test; DSST, digit symbol substitution test; IPR, income-to-poverty ratio; IRT, immediate recall test; N,number of patients; TCT, three cognitive tests.

### 3.2 Association Between Conicity Index and Cognitive Performance

**Fig. 2.**
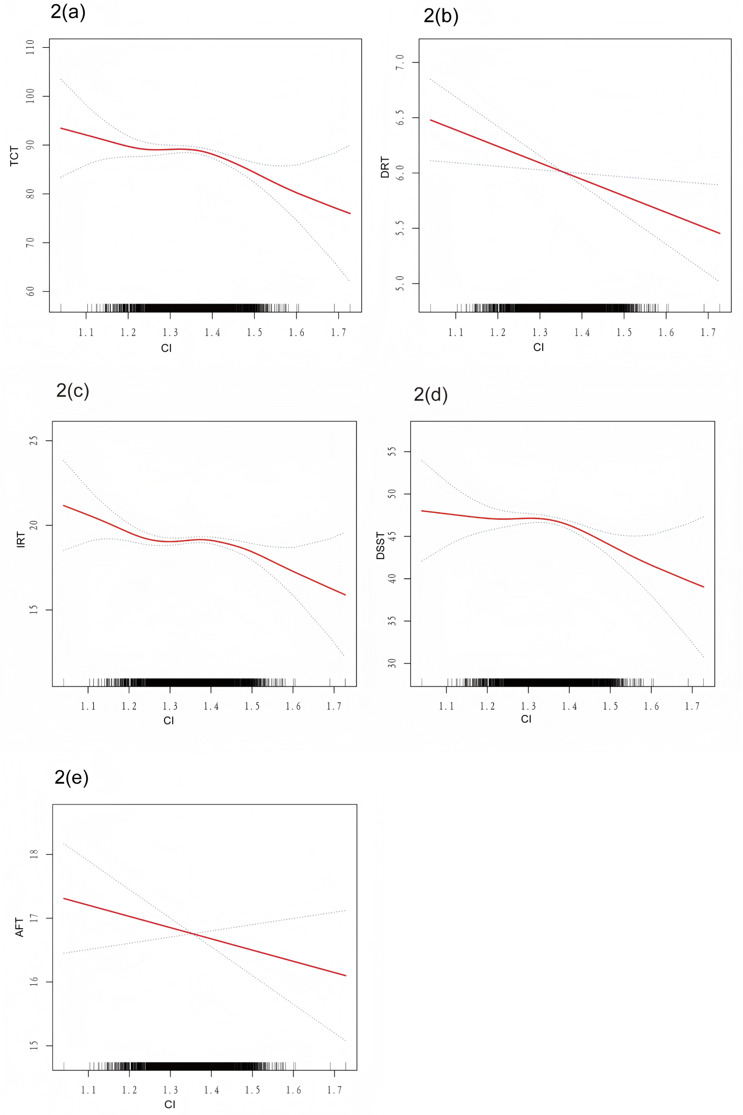
**Smooth curve fitting for conicity index and low cognitive performance**. (a) Related to TCT. (b) Related to DRT. (c) Related to IRT. (d) Related to DSST. (e) Related to AFT. Legend: Solid red lines represent the smooth curve fit between the variables, while blue bands indicate the 95% CI derived from this fit.

An increasing conicity index was associated with declining CF. In the comprehensive adjusted model (Model 3), a one-unit rise in conicity index corresponded to a 16.35-point reduction in TCT scores [β = –16.35, 95% confidence interval (CI): (–26.68, –6.02), *p* = 0.0019], a 1.52-point decrease in DRT scores [β = –1.52, 95% CI: (–2.74, –0.30), *p* = 0.0151], a 2.93-point decrease in IRT scores [β = –2.93, 95% CI: (–5.37, –0.48), *p* = 0.0190], a 2.03-point decrease in AFT scores [β = –2.03, 95% CI: (–4.88, 0.82), *p* = 0.1636], and a 9.88-point decrease in DSST scores [β = –9.88, 95% CI: (–17.11, –2.65), *p* = 0.0075]. However, no significant negative correlation was found between the conicity index and AFT scores (Table 2). The smooth curve fitting further confirmed the negative association between the conicity index and CF (Fig. 2).

**Table 2.** **Link between the conicity index and low cognitive performance**.

Cognitive function		Model 1 β (95% CI)	*p* value	Model 2 β (95% CI)	*p* value	Model 3 β (95% CI)	*p* value
TCT							
	Conicity index	–35.18 (–46.54, –23.81)	<0.0001	–16.84 (–25.77, –7.92)	0.0002	–16.35 (–26.68, –6.02)	0.0019
DRT							
	Conicity index	–2.93 (–4.00, –1.86)	<0.0001	–0.72 (–1.75, 0.31)	0.1695	–1.52 (–2.74, –0.30)	0.0151
IRT							
	Conicity index	–6.26 (–8.41, –4.12)	<0.0001	–2.14 (–4.19, –0.08)	0.0415	–2.93 (–5.37, –0.48)	0.0190
AFT							
	Conicity index	–1.44 (–4.01, 1.13)	0.2712	–1.61 (–4.00, 0.77)	0.1853	–2.03 (–4.88, 0.82)	0.1636
DSST							
	Conicity index	–24.54 (–32.51, –16.57)	<0.0001	–12.37 (–18.65, –6.09)	0.0001	–9.88 (–17.11, –2.65)	0.0075

Model 1, did not adjust for any confounders. Model 2, adjusted for age, gender, race, education level and, marital status. Model 3, adjusted for age, gender, race, education level, marital status, IPR, BMI, smoking status, alcohol consumption, hypertension, and diabetes. CI, confidence interval.

To evaluate the consistency of a link between the conicity index and cognitive decline, subgroup analyses and interaction tests were executed, categorized by gender, age, education level, race, marital status, IPR, BMI, smoking status, alcohol consumption, hypertension, and diabetes. As shown in Table 3, non-Hispanic whites and Mexican American displayed a more pronounced inverse correlation between the conicity index and TCT, AFT, and DSST scores relative to other ethnic cohorts (*p* < 0.05). No other stratifications demonstrated significant effects on the link between the conicity index and CF.

**Table 3.** **Subgroup analysis of the associations between conicity index and cognitive performance**.

Subgroup		TCT		DRT		IRT		AFT		DSST	
		β (95% CI) *p* value	*p* interaction	β (95% CI) *p* value	*p* interaction	β (95% CI) *p* value	*p* interaction	β (95% CI) *p* value	*p* interaction	β (95% CI) *p* value	*p* interaction
Gender			0.1413		0.5059		0.5978		0.0709		0.2004
	Male	–19.2 (–35.8, –2.6) 0.0236		–1.8 (–3.4, –0.2) 0.0244		–4.0 (–7.1, –0.8) 0.0131		0.8 (–3.0, 4.6) 0.6702		–14.3 (–25.9, –2.6) 0.0164	
	Female	–36.4 (–52.1, –20.60) <0.0001		–2.5 (–4.0, –1.1) 0.0008		–5.1 (–8.1, –2.2) 0.0007		–4.0 (–7.6, –0.4) 0.0296		–24.8 (–35.8, –13.7) <0.0001	
Age			0.6835		0.6232		0.2737		0.8146		0.6751
	60–69	–21.5 (–36.4, –6.6) 0.0047		–1.6 (–3.0, –0.2) 0.0228		–4.1 (–7.0, –1.3) 0.0044		0.3 (–3.2, 3.7) 0.8830		–16.0 (–26.5, –5.5) 0.0030	
	70–79	–31.5 (–52.5, –10.6) 0.0032		–2.8 (–4.8, –0.8) 0.0052		–7.0 (–11.0, –3.0) 0.0007		1.5 (–3.4, 6.3) 0.5510		–23.2 (–38.1, –8.4) 0.0022	
	≥80	–18.4 (–46.1, 9.2) 0.1911		–1.8 (–4.5, 0.8) 0.1666		–1.7 (–7.0, 3.5) 0.5206		–1.1 (–7.4, 5.2) 0.7339		–13.8 (–33.3, 5.8) 0.1681	
BMI			0.5091		0.3751		0.2975		0.7199		0.3216
	<25	–52.2 (–72.0, –32.5) <0.0001		–4.9 (–6.8, –3.0) <0.0001		–10.6 (–14.3, –6.9) <0.0001		–1.3 (–5.8, 3.2) 0.5657		–35.4 (–49.2, –21.5) <0.0001	
	≥25, <30	–43.0 (–64.4, –21.6) <0.0001		–3.0 (–5.0, –1.0) 0.0038		–6.3 (–10.4, –2.3) 0.0022		–3.7 (–8.6, 1.1) 0.1316		–30.0 (–45.0, –14.9) <0.0001	
	≥30	–34.2 (–57.5, –10.9) 0.0041		–3.7 (–5.9, –1.5) 0.0009		–7.9 (–12.3, –3.5) 0.0004		–3.6 (–8.9, 1.7) 0.1816		–19.0 (–35.4, –2.6) 0.0229	
Race			0.0010		0.4668		0.0993		0.0049		0.0008
	Mexican American	–61.8 (–97.4, –26.2) 0.0007		–3.4 (–7.0, 0.1) 0.0560		–8.1 (–15.2, –1.1) 0.0243		–11.3 (–19.5, –3.1) 0.0069		–38.9 (–63.6, –14.3) 0.0020	
	Other Hispanic	–30.7 (–71.8, 10.5) 0.1445		–2.1 (–6.2, 2.0) 0.3081		–3.3 (–11.5, 4.9) 0.4276		–5.2 (–14.7, 4.4) 0.2881		–20.1 (–48.6, 8.5) 0.1682	
	Non-Hispanic White	–66.8 (–82.2, –51.4) <0.0001		–3.8 (–5.4, –2.3) <0.0001		–9.6 (–12.7, –6.6) <0.0001		–7.5 (–11.1, –3.9) <0.0001		–45.8 (–56.5, –35.1) <0.0001	
	Non-Hispanic Black	–11.1 (–33.1, 11.0) 0.3239		–1.4 (–3.5, 0.8) 0.2227		–3.2 (–7.6, 1.2) 0.1518		–1.0 (–6.1, 4.1) 0.7027		–5.5 (–20.8, 9.7) 0.4776	
	Other race	–31.9 (–68.5, 4.7) 0.0880		–3.1 (–6.7, 0.5) 0.0949		–2.9 (–10.2, 4.4) 0.4337		7.5 (–1.0, 15.9) 0.0838		–33.3 (–58.7, –7.9) 0.0101	
Education			0.2329		0.2418		0.9588		0.8505		0.0658
	Below high school	–12.1 (–32.2, 8.0) 0.2367		–1.3 (–3.5, 0.8) 0.2158		–5.0 (–9.3, –0.8) 0.0196		–0.7 (–5.7, 4.3) 0.7853		–5.1 (–19.0, 8.9) 0.4792	
	High school	–30.6 (–51.0, –10.2) 0.0033		–3.9 (–6.1, –1.8) 0.0003		–5.9 (–10.2, –1.6) 0.0068		–1.2 (–6.3, 3.8) 0.6375		–19.5 (–33.7, –5.3) 0.0071	
	Above high school	–32.7 (–46.3, –19.2) <0.0001		–2.5 (–3.9, –1.1) 0.0006		–5.5 (–8.4, –2.7) 0.0002		0.4 (–2.9, 3.8) 0.8062		–25.1 (–34.5, –15.7) <0.0001	
Marital Status			0.6074		0.3103		0.9335		0.7392		0.4240
	Married	–44.1 (–59.6, –28.7) <0.0001		–4.1 (–5.5, –2.6) <0.0001		–6.7 (–9.7, –3.8) <0.0001		–1.4 (–4.9, 2.2) 0.4500		–32.0 (–42.8, –21.1) <0.0001	
	Widowed	–30.6 (–56.2, –5.0) 0.0193		–1.6 (–4.0, 0.9) 0.2081		–4.5 (–9.4, 0.4) 0.0713		–2.7 (–8.5, 3.1) 0.3613		–21.8 (–39.8, –3.8) 0.0174	
	Divorced	–19.9 (–48.5, 8.7) 0.1728		–1.7 (–4.5, 1.0) 0.2129		–6.2 (–11.7, –0.7) 0.0263		3.3 (–3.2, 9.8) 0.3161		–15.3 (–35.4, 4.8) 0.1351	
	Separated	–30.3 (–95.9, 35.4) 0.3667		–0.6 (–6.9, 5.6) 0.8451		–3.2 (–15.7, 9.4) 0.6212		–5.1 (–20.0, 9.8) 0.5033		–21.4 (–67.4, 24.7) 0.3634	
	Never married	–13.8 (–55.4, 27.8) 0.5164		–1.8 (–5.7, 2.2) 0.3795		–4.7 (–12.6, 3.3) 0.2517		–3.7 (–13.2, 5.7) 0.4411		–3.6 (–32.8, 25.6) 0.8075	
	Living with partner	–26.4 (–95.3, 42.4) 0.4520		0.2 (–6.4, 6.7) 0.9540		–10.3 (–23.5, 2.8) 0.1246		–3.4 (–19.0, 12.3) 0.6747		–12.9 (–61.2, 35.3) 0.5993	
IPR, n (%)			0.2493		0.1257		0.8079		0.5858		0.1794
	≤1.3	–15.2 (–35.5, 5.1) 0.1414		–3.2 (–5.3, –1.2) 0.0020		–4.6 (–8.7, –0.6) 0.0260		1.5 (–3.3, 6.3) 0.5404		–8.9 (–23.0, 5.2) 0.2159	
	>1.3, ≤3	–24.5 (–43.8, –5.3) 0.0126		–1.0 (–2.9, 0.9) 0.3138		–6.4 (–10.3, –2.6) 0.0011		0.1 (–4.5, 4.6) 0.9791		–17.2 (–30.6, –3.8) 0.0120	
	>3	–37.9 (–55.8, –20.0) <0.0001		–3.6 (–5.4, –1.8) <0.0001		–5.9 (–9.5, –2.3) 0.0013		–1.8 (–6.1, 2.4) 0.3954		–26.6 (–39.0, –14.1) <0.0001	
Alcohol consumption, n (%)			0.2092		0.8644		0.4890		0.8816		0.0943
	Yes	–42.5 (–56.4, –28.7) <0.0001		–3.0 (–4.3, –1.7) <0.0001		–7.1 (–9.7, –4.4) <0.0001		–1.9 (–5.1, 1.2) 0.2260		–30.6 (–40.3, –20.9) <0.0001	
	No	–27.1 (–46.9, –7.2) 0.0075		–3.2 (–5.1, –1.3) 0.0009		–5.4 (–9.2, –1.7) 0.0047		–2.4 (–6.8, 2.1) 0.3051		–16.1 (–30.0, –2.2) 0.0235	
Hypertension, n (%)			0.0945		0.1024		0.2789		0.1874		0.1480
	Yes	–23.0 (–37.6, –8.4) 0.0021		–2.0 (–3.4, –0.6) 0.0042		–5.0 (–7.8, –2.2) 0.0004		1.0 (–2.3, 4.3) 0.5359		–17.0 (–27.2, –6.7) 0.0012	
	No	–43.0 (–61.3, –24.6) <0.0001		–3.9 (–5.6, –2.1) <0.0001		–7.5 (–11.0, –4.0) <0.0001		–2.5 (–6.6, 1.6) 0.2336		–29.1 (–42.0, –16.2) <0.0001	
Diabetes, n (%)			0.1498		0.4280		0.3450		0.4016		0.1352
	Yes	–8.1 (–33.4, 17.2) 0.5291		–1.4 (–3.8, 1.0) 0.2668		–4.5 (–9.4, 0.3) 0.0650		0.8 (–4.9, 6.6) 0.7734		–3.1 (–20.8, 14.7) 0.7343	
	No	–32.9 (–46.3, –19.5) <0.0001		–3.0 (–4.3, –1.8) <0.0001		–6.5 (–9.1, –4.0) <0.0001		–0.6 (–3.6, 2.4) 0.7002		–22.8 (–32.2, –13.4) <0.0001	
	Broadline	–1.3 (–53.7, 51.1) 0.9619		–1.4 (–6.4, 3.5) 0.5693		0.6 (–9.4, 10.5) 0.9104		7.6 (–4.2, 19.5) 0.2066		–8.1 (–44.8, 28.6) 0.6672	
Smoking status, n (%)			0.6968		0.2621		0.2641		0.4565		0.8919
	Yes	–31.6 (–48.0, –15.1) 0.0002		–2.3 (–3.8, –0.7) 0.0039		–4.8 (–7.9, –1.7) 0.0025		–0.5 (–4.2, 3.2) 0.8001		–24.0 (–35.5, –12.5) <0.0001	
	No	–36.1 (–52.0, –20.2) <0.0001		–3.5 (–5.0, –2.0) <0.0001		–7.3 (–10.3, –4.3) <0.0001		–2.4 (–6.0, 1.2) 0.1829		–22.9 (–34.1, –11.8) <0.0001	

Notes: Age, gender, race, education level, marital status, IPR, BMI, smoking status, alcohol consumption, hypertension, and diabetes are adjusted.

## 4. Discussion

Research indicates that by addressing risk factors for cognitive impairment, the likelihood of developing cognitive decline can be reduced by up to 35% [14]. Thus, identifying modifiable risk factors is a crucial strategy for preventing cognitive deterioration. This study conducted a cross-sectional analysis of 2752 older individuals aged 60 and above in the United States to elucidate the link between cognitive impairment and the conicity index. Findings demonstrated that, in the completely adjusted model (Model 3), the conicity index was markedly negatively correlated with TCT, DRT, IRT, and DSST scores. This correlation persisted independently of age, gender, race, education level, IPR, BMI, smoking status, alcohol consumption, hypertension, or diabetes. However, no notable inverse link was found between conicity index and AFT scores, which may be attributed to AFT primarily assessing semantic long-term memory, whereas early cognitive decline in dementia typically manifests as episodic memory impairment [15]. Further subgroup analyses and interaction tests revealed a relatively stable negative association between the conicity index and cognitive decline across various population subgroups. Notably, non-Hispanic whites exhibited a more substantial inverse link between the conicity index and TCT, AFT, and DSST scores relative to other ethnic cohorts, suggesting that obesity may exert a greater influence on CF in non-Hispanic whites than in other racial or ethnic cohorts.

Obesity is an increasingly problematic global health issue with a rising prevalence. Previous research has identified obesity as a potential risk factor for cognitive decline. Clinical studies have shown that obese individuals tend to have reduced brain volumes, particularly in the hippocampus, which is essential for CF. Additionally, decreased levels of gray matter have been observed in brain regions such as the hippocampus, prefrontal cortex, and other subcortical areas [16]. Cheke *et al*. [17] found that obesity diminished functional activity in cortical areas related to episodic memory, including the hippocampus, angular gyrus, and dorsolateral prefrontal cortex. This may explain the lack of a significant correlation between conicity index and AFT, which primarily assesses semantic long-term memory in this study.

The mechanisms by which obesity may lead to cognitive impairment largely involve neuroinflammation, insulin resistance (IR) and gut microbiota dysregulation. First, excessive adipose tissue in obese subjects results in chronic low-grade peripheral inflammation. Inflammatory markers like tumor necrosis factor-α (TNF-α) and interleukin-6 (IL-6), secreted by adipocytes, can infiltrate the brain through various pathways, triggering neuroinflammatory responses, particularly in the hypothalamus. This may lead to synaptic remodeling, neurodegeneration, and impaired neuronal connectivity, ultimately affecting CF [18, 19, 20]. Second, obesity is frequently associated with varying degrees of insulin resistance, which leads to elevated insulin levels. Insulin plays a critical role in regulating neuro-metabolism and glucose uptake in the hippocampus and temporal lobes, influencing neurotransmitter release and reuptake (e.g., dopamine, acetylcholine), thereby enhancing cognition. However, when insulin levels become excessive in the brain, insulin-degrading enzymes prioritize insulin removal over amyloid-β clearance, allowing amyloid accumulation, which can impair CF [21, 22, 23]. Third, the composition of gut microbiota and the metabolism of aromatic amino acids can also be influenced by obesity, potentially impairing memory [24]. A animal study has shown that diet-induced obese mice exhibit increased intestinal permeability, and alterations in gut flora may impact CF through the gut-brain axis [25]. Epidemiological studies and meta-analyses have further revealed a significant link between obesity and an elevated likelihood of dementia. A meta-analysis of 19 longitudinal investigations, encompassing 58,964 individuals aged 35 to 65 years with up to 42 years of follow-up, found that obesity marked increases the risk of dementia [26]. Central obesity, in particular, has shown a strong association with poor cognitive performance [27].

Given these findings, the use of simple anthropometric measures to assess obesity could be an effective means of identifying individuals at risk for cognitive impairment. Monitoring and maintaining these measurements within normal ranges may improve screening and intervention strategies aimed at preventing cognitive decline. Currently, BMI is the most widely used indicator of obesity in epidemiological research. A study involving 6582 British individuals aged 50 and older, with a mean follow-up period of 11 years, revealed that a BMI ≥30 kg/m^2^ was linked to an elevated likelihood of dementia [28].

A large cross-sectional investigation conducted in Western China similarly found that elevated BMI increased the likelihood of cognitive decline in middle-aged men aged 50–59 [29]. However, a study has yielded opposite findings. For example, a baseline survey in 2014, followed by a 2018 reassessment of 5156 participants aged ≥65 years in China, suggested that the incidence of cognitive impairment in the obesity cohort (as defined by BMI) was lower than in the normal-weight cohort [30]. Additionally, in a study of older Indonesian individuals aged 60–65, it was found that the probability of cognitive impairment in the obese cohort (BMI ≥27.5 kg/m^2^) was reduced by 95.7% compared to the normal BMI cohort (18.5–22.9 kg/m^2^) [31]. These contradictory results may stem from differences in follow-up periods, comorbidities, or reverse causality. Moreover, BMI is calculated using only height and weight, in fails to capture fat distribution, which might contribute to inconsistent conclusions. In contrast, the conicity index, based on a two-cone principle, more accurately reflects abdominal fat deposition and increases with the proportion of abdominal adipose tissue [32, 33]. The conicity index is considered a reliable alternative indicator of obesity and has been used to predict visceral (abdominal) fat [34]. Abdominal obesity, compared to peripheral fat deposits in the thighs, buttocks, and limbs, is more strongly associated with an increased risk of cognitive impairment [35, 36]. Therefore, it is speculated that the conicity index may demonstrate a stronger correlation with CF than traditional indicators such as BMI. The study also confirmed lower cognitive function is associated with a higher conicity index. Intervention study results demonstrated the impact of weight loss on cognitive function. In animal model research, high-fat diet-induced obese mice that underwent exercise training exhibited improvements in CF, synaptic plasticity, and reduced neuroinflammation as body mass decreased [37]. Similarly, a clinical study shows the same findings, Alosco *et al*.’s [38] research found that the cognitive function of 78 bariatric surgery patients improved after surgery. In 2023, the Johns Hopkins University School of Medicine conducted a study on 35 women with a BMI ≥35 kg/m^2^ before and after weight loss surgery, who showed improvements in auditory attention and executive function and all tests of processing speed [39]. The study by Hathaway *et al*. [40] also demonstrated that cognition appears generally likely to improve following bariatric surgery. Therefore, for the older population, screening for the conicity index and cognitive function should be strengthened to improve cognitive function through targeted weight management in the early stages of cognitive decline. This is of great significance for improving the quality of life of older adults, promoting healthy aging and reducing the burden on families and society.

This study is cross-sectional and therefore is neither able to demonstrate a causal relationship between the temporal progression of cognitive impairment and the conicity index with these data, nor draw the conclusion that body surface measurement indicators change with time, there is also a lack of research on the possible influencing factors, including environment, region, diet, climate and drugs, which may limit the applicability of the results to people in different regions.

## 5. Conclusions

This study demonstrated that a higher conicity index is correlated with an elevated risk of cognitive dysfunction. The conicity index is helpful for the early detection of cognitive decline.

## Availability of Data and Materials

The data utilized for this research is openly accessible and obtainable from the website http://www.cdc.gov/nchs/nhanes/.
